# A survey on beliefs and attitudes of trainee surgeons towards placebo

**DOI:** 10.1186/s12893-016-0142-5

**Published:** 2016-04-27

**Authors:** Mathew J. Baldwin, Karolina Wartolowska, Andrew J. Carr

**Affiliations:** Oxford NIHR Musculoskeletal Biomedical Research Unit, Old Road, Oxford, OX3 7LD UK; The Botnar Institute of Musculoskeletal Sciences, Nuffield Department of Orthopaedics, Rheumatology and Musculoskeletal Sciences (NDORMS), University of Oxford, Old Road, Oxford, OX3 7LD UK

**Keywords:** Orthopaedics, Placebos, Surgery, Attitudes and Beliefs

## Abstract

**Background:**

The aim of this study was to investigate the beliefs and attitudes of trainee surgeons regarding placebo interventions, in surgical practice and in research, and to compare them to those of senior orthopaedic surgeons.

**Methods:**

An invitation to participate in an online survey was sent to all the email addresses in the members’ database of the British Orthopaedic Trainees Association (BOTA).

**Results:**

All 987 members of BOTA were invited to participate in the survey and 189 responded (19 %). The majority of trainees think that the placebo effect is real (88 %), has therapeutic benefits (88 %) and that placebo manipulations are permissible (98 %). Sixty per cent of respondents agree that placebo can be used outside of research, most commonly, to distinguish between organic and non-organic symptoms (36 %). Trainees are more likely than senior surgeons to use placebo for pain management (34 % vs. 12 %). They are mainly concerned about the risk of side effects associated with the use of placebo (80 %) and prefer placebo interventions with minimal invasiveness. Seventy-three per cent respondents would recruit patients into the proposed randomised controlled surgical trial.

**Conclusions:**

The views regarding efficacy, permissibility and indications for placebo among trainees are similar to those of orthopaedic consultants. Orthopaedic trainees regard placebo as permissible and show willingness to recruit into placebo-controlled trials. However, they seem to have limited understanding of mechanisms of placebo effect and underestimate its ubiquity.

**Electronic supplementary material:**

The online version of this article (doi:10.1186/s12893-016-0142-5) contains supplementary material, which is available to authorized users.

## Background

Surgical interventions are being increasingly used and the number of surgical procedures performed rises each year [1, 2]. Technological advances have created new possibilities for surgical intervention. Yet, unlike drug products, verifying the efficacy of new procedures is not currently mandatory [3]. The issue of true efficacy is particularly important in case of surgical interventions aimed primarily at improving “soft” outcome measures, such as quality of life, pain or function, as these subjective measures are prone to bias from the placebo effect; unlike “hard” outcomes, such as mortality or laboratory measures [4].

The placebo effect refers to an improvement in clinical symptoms or patients’ well-being in response to placebo manipulations such as an inactive substance or a procedure that simulates an active therapy, but itself has no specific effects [5]. The placebo effect is a consequence of patients’ expectations, verbal and non-verbal suggestions by the treating health professional as well as classical conditioning [5, 6]. An actual placebo pill or procedure is not necessary and even a positive consultation or a definitive diagnosis may have a placebo effect, with more invasive treatments potentially exerting a larger effect [7–9].

The role of placebo and placebo interventions in routine clinical practice remains controversial [10–12]. The majority of physicians admit that they have used placebo or non-specific treatments [13], but there is little known about the attitudes of surgeons towards placebo use in their speciality. Earlier studies suggested that surgeons have more defensive attitude than other specialties when it comes to including their procedures into a definition of placebo [14]. A recent survey [15] demonstrated that consultant British orthopaedic surgeons generally agree with the provided definition of placebo and believe that placebo in surgery is permissible (96 %), especially in the context of research. They believe that the placebo effect is real (92 %) and it has therapeutic effects (77 %). Importantly, over half (58 %) recognise that surgical interventions may contain a significant placebo component. However, 42 % responded that they have never performed a procedure that could have a significant element of placebo. The survey also demonstrated that surgeons have limited understanding of the mechanisms underlying the placebo effect and underestimate its ubiquity. It is important to note that this survey was conducted among surgeons who were aware of a placebo-controlled trial being currently undertaken in the UK, which could have affected their responses. Additionally, it is not known whether these attitudes represent the beliefs of the whole orthopaedic community or are a generational phenomenon. Understanding the attitudes of surgeons towards placebo may help to answer the question why there have been so few placebo-controlled trials of interventional procedures [15].

The aim of this study was to investigate the attitudes and beliefs of British orthopaedic trainee surgeons towards placebo in the context of surgical research as well as clinical practice.

## Methods

### Participants

Surgical members of the British Orthopaedic Trainees Association (BOTA) were invited via email to participate in an online survey. Although not all of the 1,111 nationally registered UK orthopaedic trainees will be BOTA members, this email database was identified as the most complete contact list available to the authors. The data collection was open between 28th July and 28th August 2014. A reminder was sent via email at the midpoint of collection. Permission for use of the email database was granted by the BOTA. This study was reviewed by the National Institute for Health Oxford Musculoskeletal Biomedical Research Unit Board at the Nuffield Department of Orthopaedics, Rheumatology and Musculoskeletal Sciences, which decided it did not require a review by an external ethics committee as it was a survey of healthcare professionals and the participation as well as the answers were fully anonymous and voluntary.

### Survey instrument

A questionnaire used in this study was derived from previously published surveys to make the results comparable with earlier studies [15–18]. Participants were asked about their age, gender, number of years since graduation, stage of their training as well as the number of patients they treat weekly. To assess the degree of exposure to research ethics and methodology we asked about their attainments of higher degrees, Good Clinical Practice (GCP) accreditation, proportion of time spent in research and the number of patients they recruited into a randomised controlled trial (RCT) within the last 12 months.

To address the controversies surrounding definitions of placebo, the survey started with three separate definitions and closed questions on whether the participants agreed with each of them. Two further closed questions asked whether the respondents believed that the placebo effect is real, i.e., has a scientific basis, and whether it has a therapeutic effect. The next three questions were related to clinical situations, in which the trainees would consider a placebo, the concerns related to placebo use as well as understanding of mechanisms underlying the placebo effect. Participants were also asked how often they performed or observed operations that they believed to have a significant placebo component. The section ended with a direct question on the participants’ overall position concerning the use of placebo in clinical practice.

The next section sought to investigate attitudes toward placebo in clinical research; a number of statements were posed and each response was rated on a 5-point Likert scale ranging from “strongly disagree” to “strongly agree”. Finally, respondents were asked to consider a scenario in which an old and new procedure co-existed, with disagreement over their efficacy and superiority. The respondents were asked to choose the design that they believed is superior from the scientific perspective. The next question asked whether they would personally recruit into a surgical trial with a placebo arm. Finally, we assessed whether acceptability is affected by the degree of invasiveness of the placebo. Several study designs, with varying physical invasiveness, were described and participants were asked to choose the studies they would personally recruit into.

The survey document is in Additional file 1. The survey was piloted on 11 orthopaedic trainees within NDORMS to ensure face validity.

### Data analysis

Participants entered their responses directly into an online survey (http://www.surveymonkey.net)

The data were summarised using percentages to describe the responses to each question. A multivariate logistic regression was used to explore the relationship between willingness to recruit into a placebo-controlled surgical trial and respondents’ characteristics. For the purposes of analysis, this outcome was reduced to a binary outcome: “would recruit” or “would not recruit”. Characteristics tested included; age, gender, training grade, time since graduation, work load, hospital type, research experience, higher degrees achieved, number of patients recruited into an RCT, GCP accreditation, belief in placebo effect, belief in therapeutic benefit of placebo, frequency of placebo use, and belief in scientific importance of placebo controls. Questions that overlapped with a previous survey of Orthopaedic consultants [15] (attitudes on placebo permissibility, indications for utilising placebo, placebo mechanisms, frequency of placebo use, belief in the placebo effect and belief in the therapeutic benefit of placebo) were compared using a *χ*^2^ analysis. All analysis was conducted with SPSS (Version 21).

## Results

### Respondent characteristics

There were 987 email addresses in the BOTA database but only 189 members participated in the survey and 152 of them completed the whole questionnaire, a response rate of 19 %. The respondents were most male (83 %), aged between 30 and 39 (77 %), and between 3^rd^ and 5^th^ year of speciality training (46 %). The majority worked in a district general hospital (51 %), and treated 31 to 50 patients per week (48 %) (Table 1). Most participants had another higher degree, in addition to their primary medical degree (79 %), but only 31 % were involved in research on a weekly basis. Most (75 %) had not recruited a patient into a RCT within the last 12 months (Table 2).

**Table 1:** Sample characteristics

Category	N	Count (%)
Age	189	
20–29		25 (13)
30–39		146 (77)
≥40		18 (10)
Gender	189	
Male		156 (83)
Female		33 (17)
Years Since Graduation	189	
0–5		25 (13)
6–10		103 (55)
11–15		51 (27)
≥16		10 (5)
Patients seen each week	189	
<10		0 (0)
10–30		31 (16)
30–50		91 (48)
>50		67 (36)
Training grade	189	
Locum Appointed for Training		15 (8)
Speciality Registrar (ST 3–5)		86 (46)
Speciality Registrar (ST 6–8)		67 (35)
Fellow		13 (7)
Non-training grade Registrar		8 (4)

**Table 2:** Research experience

Category	N	Count (%)
Research Involvement	189	
2–5 days/week		14 (7)
1 day/week		46 (24)
1–3 days/month		70 (37)
<1 day/month		59 (31)
Additional Higher Degrees	189	
BSc or BA		58 (31)
MSc, MA, MPhil or MS		60 (32)
MD, PhD, DPhil or DSc		31 (16)
None		40 (21)
GCP certification^a^	189	
Yes		82 (43)
No		107 (57)
Patients recruited into RCT^b^	189	
None		142 (75)
1–5		26 (14)
6–10		11 (6)
>11		10 (5)

^a^Good Clinical Practice (GCP)

^b^ Randomised Controlled Trial (RCT) within last 12 months

### Definitions of placebo

**Fig. 1:**
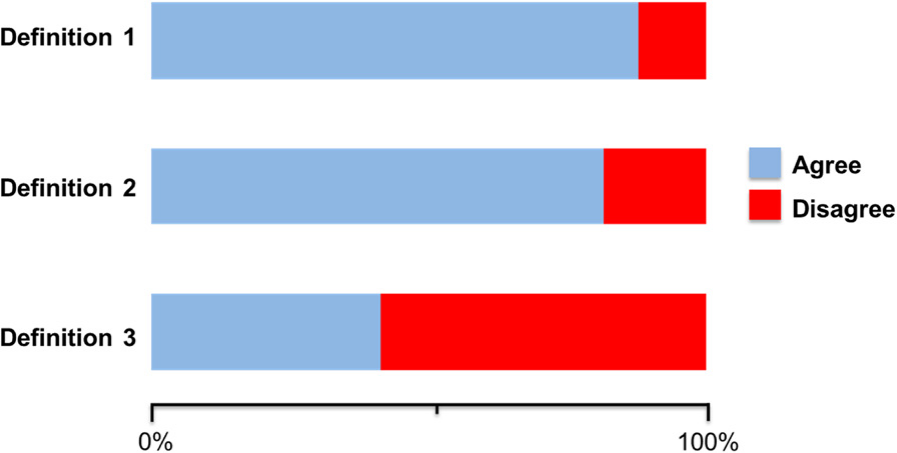
Definitions of placebo. Respondents were asked about their position: “Agree” or “Disagree” regarding three different definitions of placebo. The definitions are listed on the y-axis and the cumulative @percentage of respondents are listed on the x-axis. See body of manuscripts for wording of each definition

The majority of participants (88 %) agreed with the definition of placebo as “any intervention or treatment, that objectively is known to have no specific effect, but for which a beneficial outcome occurs as a result of the patient’s belief in its efficacy” [Definition 1] (Fig. 1). Ten respondents provided additional comments: nine felt the definition described placebo effect and not placebo, since the later could also be harmful and one respondent disagreed with describing a placebo as a “treatment”.

Slightly fewer participants (81 %) agreed with the definition of placebo for surgical practice, i.e., “an intervention where patients undergo a surgical procedure that has the appearance of a therapeutic intervention, but during which the essential therapeutic manoeuvre is omitted” [Definition 2]. Of the 35 respondents who disagreed nine provided comments, including four who felt it should be called a “sham surgery” and not a “placebo”, two participants commented that the definition was too narrow, i.e., surgical practice was not synonymous with surgical intervention, two who disagreed purely on ethical grounds, and one respondent commented on the fact that the “essential manoeuvre” is often difficult to determine.

Our final definition explored whether respondents felt a definition of placebo “should include therapies which are given by a surgeon in the belief that they are effective and specific even though, and unknown to the surgeon, they are in fact non-specific” [Definition 3]. This option was the most controversial and 59 % of respondents disagreed with the given definition. Six participants expanded on their reasons for disagreement: three expressed a fundamental objection, stating that if a surgeon believes in the efficacy, it cannot be classified as placebo, and further three respondents noted that disagreement over the efficacy of many procedures exists rendering the definition too broad i.e., too many therapies could be classified as placebo.

### Beliefs about placebo and indications for its use

Most of the respondents believed that the placebo effect was real and had a scientific basis (88 %), with a similar proportion believing placebo could provide therapeutic benefit to patients (88 %); these two factors were associated (*χ*^2^ = 7.66, df = 1, *p* = 0.006) and were not significantly different from the views of consultants.

When asked about the circumstances, in which they would consider using a placebo procedure, 40 % of trainees answered that they would not use placebo outside of research. The most common scenario, in which respondents would consider using placebo, was as a diagnostic tool to distinguish between organic and non-organic symptoms (36 %) as well as to control pain (34 %). Fourteen per cent would consider using placebo to mollify a complaining patient and 7 % would use it to maintain a good doctor-patient relationship (Table 3). There was a trend toward a greater use of placebo to control pain among trainees (34 % vs. 12 %, *χ*2 = 14.8, df = 1, *p* < 0.001), with no difference between trainees and senior surgeons for all other scenarios.

**Table 3:** Opinions regarding placebo use, mechanisms and concerns

Question	N	Responded Yes (%)
In which on the following situations have you considered placebo? (more than one option allowed)	180	
As a diagnostic tool		64 (36)
When all other therapies have been exhausted		50 (28)
As a treatment for a non-specific symptoms		44 (24)
Instead of surgery, when using surgery was not justified		39 (22)
As a supplement to surgery		34 (19)
To calm a patient or mollify a complaining patient		25 (14)
To control pain		61 (34)
To maintain a good relationship with a patient		12 (7)
Never as a formal treatment option outside of research		71 (40)
What are your concerns regarding the use of placebo in surgery? (more than one option allowed)	176	
It involves deception		104 (59)
It endangers patient-surgeon trust		96 (55)
Because of legal problems		68 (39)
Because of possible side effects		52 (30)
It is ineffective		23 (13)
None of the above		22 (13)
In your opinion what is the mechanism behind the placebo effect? (more than one option allowed)	180	
Psychological		166 (92)
Unexplained Factors		68 (38)
The natural course of a disease		72 (40)
Conditioning		40 (22)
Physiological		36 (20)
Positive Energies		5 (3)
Other		6 (3)

### Concerns over placebo use

The main concerns expressed by trainee surgeons were similar to those of senior surgeons: the main two issues were that placebo involved deceiving the patient (59 %) and risked damaging patient-surgeon trust (55 %). Reservations over legal implications (39 %) and potential side-effects (30 %) were also commonly expressed. Only 13 % of trainees would not use placebo because they felt it was ineffective (Table 3).

### Mechanism of the placebo effect

Most participants (92 %) answered that the placebo effect is a result of psychological mechanisms but only 22 % recognised the role of conditioning and only 20 % responded there is a physiological basis for the placebo effect (Table 3). Forty per cent responded that the placebo effect was a consequence of the natural history of a disease and 38 % believed that the placebo effect is the consequence of as yet unexplained factors. These beliefs were not significantly different from those expressed by senior surgeons.

### Frequency of the placebo effect in surgery

Only twenty-one per cent of participants reported that they had never observed an operation that they believed to have a significant placebo component. In comparison, 42 % of senior surgeons answered that they had never performed a procedure that they believed had a significant placebo component (21 % vs. 42 %, *χ*2 = 11.9, df = 1, *p* < 0.001). Other trainees reported observing such procedures infrequently: about once a year (36 %) or less than one case per year 24 %. Only 1.8 % felt they observed operations with placebo components on a weekly basis.

### Attitudes toward permissibility of placebo

Attitudes towards permissibility of placebo, in general, among the trainees were not significantly different from the previously reported opinions of orthopaedic consultants. When asked whether the use of placebo is permitted if patients gave informed consent, only 2.2 % of trainees felt that placebo should always be prohibited. Over a third believed that placebo should only be used in clinical research trials (36 %), whereas 22 % responded that placebo could be used in both clinical practice and clinical research trials. Twenty- nine per cent responded that placebo could be used in clinical practice if research supported its evidence. However, fewer (11 %) were comfortable with the use of placebo in clinical practice if the supporting evidence was only based on prior experience, within the department or personal.

### Placebo in clinical research

**Fig. 2:**
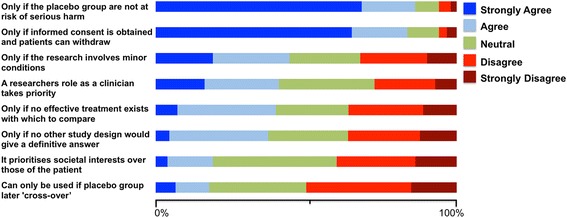
Attitudes toward placebo in clinical research. Respondents were asked to rate their position regarding a number of statements concerning the use of placebo in clinical research on a 5-point scale with anchors from “Strongly Agree” to “Strongly Disagree”. Statements are listed on the y-axis and the cumulative percentage of respondents on the x-axis

Participants were asked about their opinion regarding commonly expressed issues on the use of placebo in clinical research (Fig. 2). The most unanimous viewpoint, with over 80 % agreement, was that placebo can only be used if informed consent is sought, and if patients in the placebo group are not at risk of serious harm. Respondents’ views other topics were split, for example, 40 % disagreed and 41 % had a neutral stance on whether using a placebo arm prioritises the interests of society over those of an individual.

### Acceptability of placebo

Out of 152 trainees who answered this question, 47 % preferred a design comparing new technique with old and 40 % favoured a placebo-controlled study. The remaining 13 % would rather use an alternative design; most often a trial including all three procedures; although one respondent suggested two separate trials comparing each technique, old and new one, to placebo.

**Fig. 3:**
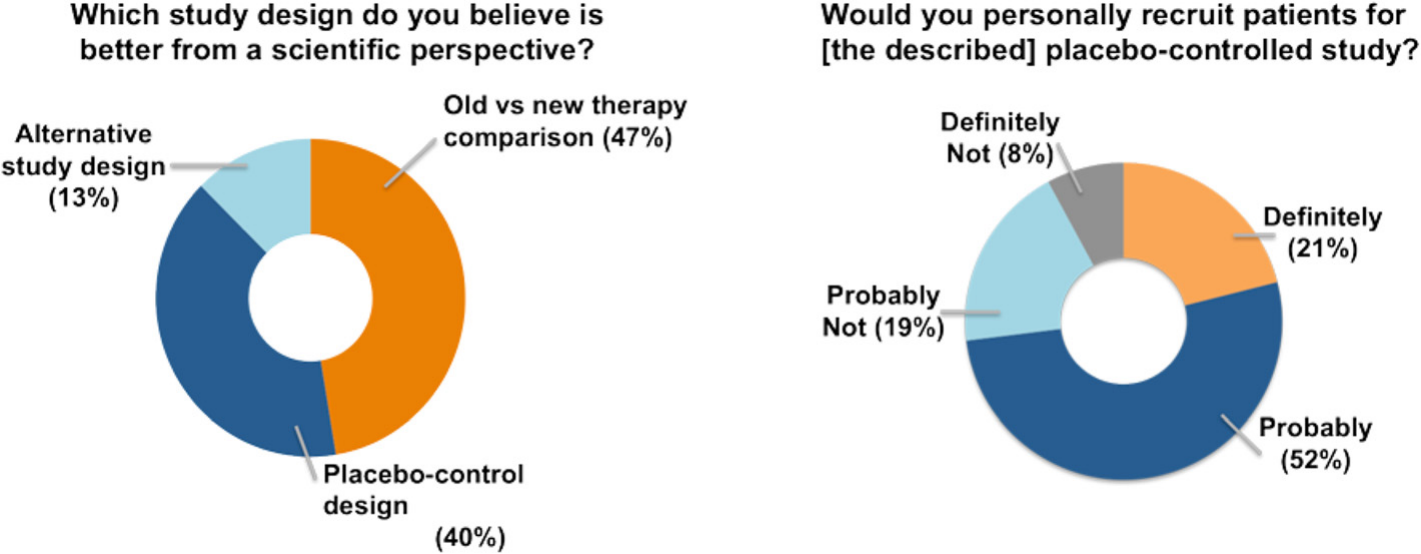
Scientific acceptability of placebo and willingness to recruit. Please note old versus new therapy comparison refers to an active-control design

At the practical level, 73 % of trainees responded that they would recruit patients into a proposed trial. The trainees who preferred the design comparing new treatment with a placebo would “definitely” (30 %) or “probably” (56 %) recruit patients into such a trial (Fig. 3). Out of respondents who chose the old versus the new design, only 11 % would definitely recruit, and 51 % would probably recruit into it, whereas 13 % would not recruit into such a trial. Interestingly, four of respondents who suggested an alternative design, with both interventions and placebo, would not recruit into it.

### Placebo invasiveness

We also investigated whether acceptance of placebo surgery depends on the invasiveness of the placebo procedure. Responses were split along the line of whether the procedure should be arthroscopic or involve open surgery (Table 4). Several participants (11 %) answered that they would not recruit and expressed strong ethical objections against recruiting into any form placebo-controlled surgical trial.

**Table 4:** Invasiveness of placebo control

Survey Question	N	Responded Yes (%)
Would you personally recruit patients into a placebo controlled trial if the placebo group undergo: (you may choose more than one answer)	152	
Skin incisions only, sufficient to imitate arthroscopic surgery		89(59)
Skin incisions only, sufficient to imitate open surgery		37(24)
Incisions sufficient to enable arthroscopic exposure and inspection of the joint		73(48)
Incisions sufficient to enable open exposure and inspection of the joint		20(13)
I would not recruit		17(11)

### Willingness to recruit

Willingness to recruit into a placebo-controlled surgical trial was associated with a belief in the therapeutic effect of placebos (Odds Ratio [OR] 4.85, 95 % Confidence Interval [CI] 1.36–17.2), frequent observation of operations with a placebo component (OR 10.3, 95 % CI 1.97–53.5) and a belief in the scientific importance of placebo controls (OR 5.17, 95 % CI 1.74–15.3).

## Discussion

### Summary of main findings

The majority of orthopaedic trainees recognise that surgical procedures can be classified as a placebo but fewer realise that the placebo effect can be created unintentionally. Most find the use of placebo in clinical practice ethically acceptable. Deception and the associated damage to trust are the main concerns over the use of placebo. Most trainees believe the placebo effect has real and therapeutic effects, explained largely through psychological mechanisms. Considerably fewer would translate their beliefs into clinical practice. The main reason why trainees would consider using placebo would be to distinguish organic from non-organic symptoms or to manage pain.

In the context of research, the importance of protecting an individuals’ right to make informed choices and the need to minimise risk of harm are the most commonly expressed ethical concerns. The scientific validity of placebo is recognised and this equated with willingness to recruit patients.

### Differences in results in comparison to other surveys

There is no generally accepted definition of placebo [14, 16]. In our survey most respondents agreed with a definition of placebo as “beneficial treatment that is known to have no specific effect” and specifically accepted that surgical procedures can have a placebo effect. This is similar to the responses of senior orthopaedic surgeons [15] but this is in contrast to the earlier study that observed that surgeons did not believe that surgical procedures could be regarded as placebo because they had a strong therapeutic effect [14].

Physician’s personal expectations [19] as well as positive consultations and suggestions can generate a placebo effect [7] and this seems to be appreciated among physicians [18]. In contrast, fewer orthopaedic trainees demonstrate understanding of these concepts. Only 41 % would include in a definition of placebo “therapies believed by the surgeon to be effective and specific even though, and unknown to the surgeon, they are in fact non-specific”. While the views of orthopaedic surgeons toward placebo are not as restrictive as those described before [14] but it seems that they remain conservative relative to other specialities.

The respondents would recruit more willingly into studies with minimally-invasive, arthroscopic, procedures rather than into open surgery. Indeed, as the invasiveness of the placebo procedure increases, there is a corresponding fall in the proportion of trainees willing to recruit patients. When specifically asked about their concerns about placebo in research, the most widely held beliefs are that risks to the participant must be minimised and informed consent granted. The attitudes and concerns of surgeons may explain, at least to some degree, why the vast majority of existing placebo-controlled surgical trials investigated minimally-invasive procedures [20]. It is important to note that some of the respondents in this survey appreciated the values of the three-arm design, i.e., of comparing two surgical procedures head to head as well as to a placebo intervention; however, they would still not recruit into such a trial.

Interestingly, in the context of clinical practice, trainees express a greater level of concern towards patient deception and subsequent damage to the doctor-patient relationship rather than potential side effects of placebo use. Trainees are aware of the need for informed consent and minimising harm so the obligations placed on them by the Declaration of Helsinki and medical regulatory bodies. Respondents’ opinions on other ethical issues, which are often a subject of a heated ethical debate, are divided.

The majority of our respondents believe that the placebo effect is real and that is has therapeutic benefits, which is similar to our previous survey of orthopaedic consultants [15] as well as to previous studies which demonstrated that most doctors believe the placebo effect is real (68–95 %) and can produce a therapeutic benefit (68–96 %) [16, 21, 22].

The circumstances in which placebos are used are complex. Among community physicians the most commonly reported reasons are to treat non-specific symptoms or to calm a patient [18]. In the hospital settings, it is more often used to alleviate pain or anxiety [16, 17] and as a tool to distinguish between organic and non-organic symptoms. [17, 23] Orthopaedic consultants and trainees in the UK are most likely to use placebo as a diagnostic tool, as treatment for non-specific symptoms or when all the other therapies have been exhausted [15]. The only significant difference is a greater use of placebo to control pain among trainees. It is surprising that both groups believe that only non-organic symptoms may possibly improve after placebo treatment.

Orthopaedic trainees commonly attribute the mechanism of the placebo effect to psychological factors. Our findings are similar to the previously reported beliefs among orthopaedic consultants as well as physicians [15, 16]. Both cohorts recognise the role of psychological factors but underestimate the role of conditioning. However, only one in five respondents understands the importance of conditioning and the fact that the placebo response involves actual physiological changes. Confusion also exists over how the placebo effect differs from the natural history of a disease [5] and between the true placebo effect, i.e., specific to the placebo manipulations, and the overall change in the placebo arm. None of the respondents suggested adding an observational, non-interventional group, to control for the natural history of disease. This is concerning as without an observational group it is not possible to separate a placebo response into the true placebo effect and non-specific effects [4]. Furthermore, the absence of a observational arm results in no baseline from which to evaluate the harms and benefits of the surgical procedure [20]. However, placebo-controlled surgical randomised controlled trials tend not to include an observational group [20]. It is also concerning that over one third of trainees replied that the placebo effect is caused by unexplained phenomena.

Most doctors find the use of placebo in clinical practice acceptable but only a minority have no reservations. Permissibility depends on the type of placebo and the circumstances of its use [13]. Evidence supporting placebo improves acceptability, with clinical research being a more powerful determinant than personal or departmental experiences alone [15, 16]. Surgeons report using procedures with a possible placebo element less often than physicians [13, 15]. This may be related to a more direct involvement in treatment [24] or it may reflect the same phenomenon that was reported by Shapiro and Struening [14], namely, that surgeons acknowledge existence of placebo effect but are unwilling to admit that some of the effect of surgical procedure may be associated with a placebo effect. It is interesting that, compared to senior surgeons, fewer trainees reported that they have never observed an operation with a placebo component [15].

### Strengths and limitations of this study

The participants were representative of the trainees in the UK. The responses were generated from all stages of training and the proportion of males to female (17.5 %) was also similar to the gender split among current trauma and orthopaedic registrars [25].

The main limitation of this survey is that the response rate cannot be accurately calculated as it is not known how many trainees in the BOTA database actually received the invitation to participate. While a low response rate could be related to the delicate nature of the topic, the rate was smaller than in the other surveys (46–57 %) [14, 19, 23] and lower than in the survey of senior orthopaedic surgeons (51 %) [14]. It may be that trainees are less likely to participate in survey research, perhaps owing to time pressures or survey fatigue. Additionally, it should be noted that the survey of senior surgeons [15] was distributed during a research conference, which might have primed participants to the importance of research and inflated response rates.

## Conclusions

There may be an increasing awareness of the frequency of placebo use amongst junior orthopaedic surgeons. However, other than a greater belief in the role of placebo in pain management, attitudes between trainees and consultants are highly comparable. Most orthopaedic trainees believe that the placebo effect is real but its mechanisms of actions and its pervasive nature remain underappreciated. Without an understanding that the placebo effect is a component of every medical treatment, the importance of placebo controlled surgical trials cannot be properly understood. High quality research that changes practice and improves care can only be successfully undertaken if there is a general support of the orthopaedic community. Providing trainees with better training in research methodology, so that they understand the strengths and limitations of different trial designs, may facilitate this process.

### Ethics approval and consent to participate

This study was reviewed by the National Institute for Health Oxford Musculoskeletal Biomedical Research Unit Board at the Nuffield Department of Orthopaedics, Rheumatology and Musculoskeletal Sciences, which decided it did not require a review by an external ethics committee as it was a survey of healthcare professionals and the participation as well as the answers were fully anonymous and voluntary.

### Availability of supporting data

The datasets supporting the conclusions of this article are included within the article.

## Additional file

Additional file 1: The survey document. (PDF 285 kb)

## Abbreviations

95%CI: 95 % confidence interval
BOTA: British Orthopaedic Trainees Association
Df: degrees of freedom
NDORMS: Nuffield Department of Orthopaedics, Rheumatology and Musculoskeletal Sciences
OR: odds ratio
RCT: randomised controlled trial
SPSS: statistical package for the social sciences
UK: United Kingdom
