# Supplementary material for: A survey on beliefs and attitudes of trainee surgeons towards placebo
Source: BMC Surg. 2016 Apr 27;16:27. doi: 10.1186/s12893-016-0142-5 (PMC4847348; doi:10.1186/s12893-016-0142-5)
Supplement: Additional file 1: — The survey document. (PDF 285 kb) [file 12893_2016_142_MOESM1_ESM.pdf]

Demographics

*1. Which category below includes your age?

- 20-29
- 30-39
- 40-49
- 50-59
- 60+

*2. What is your gender?

- Female
- Male

*3. Which of the following best describes your level? (you may choose more than one option)

- Core Surgical Trainee
- Locum Appointed for Training (LAT)
- Speciality Registrar (ST 3-6)
- Speciality Registrar (ST 6-8)
- Speciality Registrar (non-training post)
- Academic Orthopaedic Trainee
- Research Fellow
- Post CCT Fellow
- Consultant Orthopaedic Surgeon
- Not currently working in the UK
- Not currently working

Other (please specify)

*4. How many years has it been since you graduated from medical school?

- 0-5
- 6-10
- 11-15
- 16-20
- 21-25
- 26-30
- 31-35
- 36-40
- 41+

*5. Which of the following best describes the type of hospital that you are currently work in?

- District General Hospital
- Level 1 Trauma Centre
- Tertiary referral centre
- Private Practice

Other (please specify)

*6. Which of the following best describes the region in which you are working?

- East Anglia
- East Midlands
- Greater London
- North East
- Northern Ireland
- North West
- Scotland
- South East
- South West
- Wales
- West Midlands
- Yorkshire and the Humber

Other (please specify)

*7. On average how many patients do you see each week in total?

- Less than 10
- 10 to 30
- 30 to 50
- 50 to 70
- More than 70

*8. Which of following best describes your current involvement in research (excluding audit)?

- More than 5 days per week
- 2-5 days per week
- 1 day per week
- 1-3 days per month
- Less than 1 day per month

9. Have you previously undertaken any of the following degrees?

- Bachelor degree other than MBChB/MBBS
- MSc, MPhil, MS or ChM
- MD, PhD, DPhil or DSc
- No additional degree(s)

Other (please specify)

*10. How many patients have you personally recruited into any randomised clinical trial in the past 12 months?

- None
- 1-5
- 6-10
- 10-20
- More than 20

*11. Have you ever undertaken Good clinical Practice (GCP) training and been awarded a GCP certificate ?

- Yes
- No

Definition of placebo

***12. Do you agree with the following definition of placebo ?**

The term "placebo" refers to any intervention or treatment, that objectively is known to have no specific effect, but for which a beneficial outcome occurs as a result of the patient believing in its efficacy

- I Agree
- I Disagree

Additional Comments

***13. Do you agree with the following definition of placebo for surgical practice ?**

The term "placebo" refers to an intervention where patients undergo a surgical procedure that has the appearance of a therapeutic intervention, but during which the essential therapeutic manoeuvre is omitted

- I Agree
- I Disagree

Additional Comments

***14. A definition of placebo should include therapies which are given by a surgeon in the belief that they are effective and specific even though, and unknown to the surgeon, they are in fact nonspecific**

- Yes
- No

Additional Comments

The use of placebo in clinical practice

***15. Do you believe that the placebo effect is real (i.e. has a scientific basis)?**

- Yes
- No

Additional Comments

***16. Do you believe that placebo has a therapeutic benefit?**

- Yes
- No

Additional Comments

***17. In the following situations, would you ever consider the use of placebo? (you may choose more than one option)**

- As a diagnostic tool (i.e. in order to distinguish between an organic and a non-organic disorder)
- When all other therapies have been exhausted
- As a treatment for a non-specific symptom
- Instead of surgery, when undertaking surgery was not justified
- As a supplement to surgery
- To calm a patient or to mollify a complaining patient
- To control pain
- To maintain a good relationship with a patient
- Never as a formal treatment option outside of clinical research

Additional Comment(s)

***18. What are your concerns regarding the use of placebo in surgery? (you may choose more than one option)**

- It involves deception
- It endangers patient-surgeon trust
- Because of legal problems
- Because of possible side effects
- It is ineffective
- None of the above (please specify)

*19. Scientific data suggest that some patients benefit from the placebo effect. In your opinion, what is the mechanism behind this phenomenon? (you may choose more than one option)

- ☐ Psychological
- ☐ Unexplained factors
- ☐ The natural course of the illness
- ☐ Conditioning
- ☐ Physiological
- ☐ Positive energies
- ☐ Other (please specify)

*20. Assuming that patients in receipt of placebo have given informed consent what is your view on use of placebo?

Please choice the single most appropriate answer

- ○ Always prohibited
- ○ Permitted in clinical practice if research supports its efficacy
- ○ Permitted in clinical practice if prior experience, within the department or personally, supports its efficacy
- ○ Permitted only in clinical research trials
- ○ Permitted in both clinical practice and clinical research trials

*21. How often have you used or observed operations that you believe have a significant placebo component?

- ○ Never
- ○ Rarely (<1 per year)
- ○ Occasionally (>1 per year)
- ○ Often (>1 per month)
- ○ Regularly (>1 per week)

The use of placebo in clinical research

***22. What is your position concerning the use of placebo in clinical research?**

For each statement below indicate your personal opinion according to whichever of the following is closest to your opinion.

1- Strongly disagree, 2- Disagree, 3- Neutral, 4- Agree, 5- Strongly Agree

	1	2	3	4	5
It prioritises societal interests over those of the patient	☐	☐	☐	☐	☐
A researchers role as a clinician should take precedent over their role as a scientist	☐	☐	☐	☐	☐
Can only be used if no proven effective treatment exists with which to compare the new therapy	☐	☐	☐	☐	☐
Can only be used if no other study design would provide a definitive answer	☐	☐	☐	☐	☐
Can only be used if the placebo group later 'cross-over' to receive the trial therapy or procedure	☐	☐	☐	☐	☐
Can only be used if the placebo group are not at risk of serious or irreversible harm	☐	☐	☐	☐	☐
Can only be used if informed consent is obtained and patients have the right to withdraw	☐	☐	☐	☐	☐
Can only be used for research involving minor conditions that are not life threatening	☐	☐	☐	☐	☐

Additional Comments

[ ]

***23. There is disagreement in the clinical community and conflicting scientific evidence regarding the efficacy of a new surgical procedure. Uncertainty also exists regarding the efficacy of the traditional technique. Two study designs are proposed to investigate whether the new technique is actually effective.**

1) Placebo-control study - This would compare the new technique against placebo surgery i.e. a procedure that is indistinguishable from the actual surgery but which omits the step believed to be responsible for therapeutic benefit. Participants are randomly assigned and are unaware of their group allocation

2) Active-control study - This would compare the new surgical procedure against a traditional technique. Participants are randomly assigned and are unaware of their group allocation

Which study design do you believe is better from a scientific perspective?

- ○ Placebo-control study
- ○ Active-control study
- ○ An alternative study design (please specify)

[ ]

***24. Would you personally recruit patients for the placebo-control study design in the proposed trial?**

- ○ Definitely
- ○ Probably
- ○ Probably not
- ○ Definitely not

***25. You are asked to recruit patients into a randomised clinical trial that is investigating the efficacy of rotator cuff repair. Surgical repair will be compared against a placebo intervention. Rotator cuff repair can be performed either as an open or arthroscopic procedure, currently there is conflicting evidence about the best approach.**

Would you personally recruit patients into the trial if the placebo group undergo: (you may choose more than one answer)

- ☐ Skin incisions only, sufficient to imitate arthroscopic surgery
- ☐ Skin incisions only, sufficient to imitate open surgery
- ☐ Incisions sufficient to enable arthroscopic exposure and inspection of the shoulder joint
- ☐ Incisions sufficient to enable open exposure and inspection of the shoulder joint

Additional comments
